# Neurofibrillary tangle characterization in the olfactory bulb among clinicopathologic subtypes of Alzheimer’s disease

**DOI:** 10.1002/alz.095134

**Published:** 2025-01-09

**Authors:** Jessica L Chalk, Rain Kwan, Nicholas B Martin, Christina M. Moloney, Baayla D.C. Boon, Sara R Dunlop, Darren M. Rothberg, Ashley C. Wood, Jessica F. Tranovich, Ranjan Duara, Neill R Graff‐Radford, Dennis W. Dickson, Melissa E. Murray

**Affiliations:** ^1^ Mayo Clinic, Jacksonville, FL USA; ^2^ Mount Sinai Medical Center, Miami Beach, FL USA; ^3^ Department of Neurology, Mayo Clinic, Jacksonville, FL USA

## Abstract

**Background:**

Alzheimer’s disease (AD) is neuropathologically heterogeneous and can be objectively classified along a spectrum of corticolimbic tangle distribution as hippocampal sparing (HpSp) AD, typical AD, and limbic predominant AD. The olfactory bulb is an early area of tau accumulation with a direct connection to the amygdala. Although tau pathology has been identified in the olfactory bulb, its association with AD subtypes remains unclear. To investigate these differences, tau markers were characterized within the olfactory bulb among AD subtypes.

**Method:**

The cohort included 39 AD cases (HpSp AD: n = 15, typical AD: n = 12, limbic AD: n = 12). Immunofluorescence staining was conducted on 5 µm thick formalin‐fixed paraffin‐embedded olfactory bulb sections using phosphorylated tau (AT8 & PHF‐1) and C‐terminal tau (2E9) antibodies. Slides were digitally scanned on the PhenoImager™ HT slide scanner and analyzed using QuPath Digital Analysis software, quantifying tau burden as a percentage of positive staining.

**Result:**

In whole olfactory bulbs, AT8 was observed as intrasomal tangle‐like immunoreactivity with some extrasomal neuritic staining. AT8 burden did not differ among subtypes (HpSp AD median = 0.28% [interquartile range 0.014‐1.7%], typical AD = 0.28% [0.027‐2.24%], limbic AD = 0.39% [interquartile range 0.39‐0.74%]). PHF‐1 was observed as intrasomal tangle‐like immunoreactivity with abundant extrasomal neuritic pathology. PHF‐1 was observed to be highest in limbic AD (HpSp AD = 3.5% [0.63‐11%)], typical AD = 3.2% [0.38‐13%], and limbic AD = 5.3% [1.97‐10%]) and differed from typical AD (p<0.05). 2E9 was observed as intrasomal tangle‐like immunoreactivity with minimal extrasomal staining and not found to differ among subtypes (HpSp AD = 0.18% [0.036‐2.2%], typical AD = 0.15% [0.021‐2.4%], and limbic AD = 0.29% [0.0030‐8.2%]).

**Conclusion:**

Abundant tau accumulation was observed among all AD subtypes with some suggestion of higher tau burden in limbic predominant AD. Given the direct connection between olfactory bulb and amygdala, it is interesting to speculate of an environmental contribution to limbic predominant AD through olfactory bulb damage. Future studies will expand the cohort to include AD cases with Lewy body disease co‐pathology and primary tauopathies with a focus on the anterior olfactory nucleus as the primary site of tau pathology.

